# Permeability of Novel Chitosan-*g*-Poly(Methyl Methacrylate) Amphiphilic Nanoparticles in a Model of Small Intestine In Vitro

**DOI:** 10.3390/polym10050478

**Published:** 2018-04-27

**Authors:** Imrit Noi, Inbar Schlachet, Murali Kumarasamy, Alejandro Sosnik

**Affiliations:** Laboratory of Pharmaceutical Nanomaterials Science, Department of Materials Science and Engineering, Technion-Israel Institute of Technology, 3200003 Haifa, Israel; imritnoi@gmail.com (I.N.); inbarschlachet@gmail.com (I.S.); drmuralinano@gmail.com (M.K.)

**Keywords:** Chitosan-*g*-PMMA amphiphilic nanoparticles, thiolated polymers, mucoadhesion, mucosal drug delivery, Caco-2 and HT29-MTX cell lines, apparent permeability in vitro

## Abstract

Engineering of drug nanocarriers combining fine-tuned mucoadhesive/mucopenetrating properties is currently being investigated to ensure more efficient mucosal drug delivery. Aiming to improve the transmucosal delivery of hydrophobic drugs, we designed a novel nanogel produced by the self-assembly of amphiphilic chitosan graft copolymers ionotropically crosslinked with sodium tripolyphosphate. In this work, we synthesized, for the first time, chitosan-*g*-poly(methyl methacrylate) nanoparticles thiolated by the conjugation of *N*-acetyl cysteine. First, we confirmed that both non-crosslinked and crosslinked nanoparticles in the 0.05–0.1% *w/v* concentration range display very good cell compatibility in two cell lines that are relevant to oral delivery, Caco-2 cells that mimic the intestinal epithelium and HT29-MTX cells that are a model of mucin-producing goblet cells. Then, we evaluated the effect of crosslinking, nanoparticle concentration, and thiolation on the permeability in vitro utilizing monolayers of (i) Caco-2 and (ii) Caco-2:HT29-MTX cells (9:1 cell number ratio). Results confirmed that the ability of the nanoparticles to cross Caco-2 monolayer was affected by the crosslinking. In addition, thiolated nanoparticles interact more strongly with mucin, resulting in a decrease of the apparent permeability coefficient (*P*_app_) compared to the pristine nanoparticles. Moreover, for all the nanoparticles, higher concentration resulted in lower *P*_app_, suggesting that the transport pathways can undergo saturation.

## 1. Introduction

Oral administration of medication is the most popular and preferred method for patients and physicians [1]. It is painless, safe, and enables self-administration. However, it also presents a number of drawbacks that lead to a significant decrease of the oral bioavailability. The most relevant are low physicochemical stability in the gastrointestinal tract and the presence of a mucus layer that reduces the drug absorption rate and extent into the systemic circulation [2]. 

Mucus is a viscoelastic gel mainly formed by water and the glycoprotein mucin that covers all the exposed epithelial surfaces in the body that are not covered by skin such as the respiratory system, the gastrointestinal tract, the vagina, and the eye. Mucus is a porous and semipermeable barrier that enables exchange of nutrients, water, and gases while being almost impenetrable to most pathogens and protecting the epithelium from chemical, physical, and mechanical insults [3].

The composition, properties (e.g., thickness and pH), and function of mucus change according to the organ and even its portion. In the specific case of the gastrointestinal tract, the mucus is classified into two families: the inner cell-associated (“firmly adherent”) mucus that contains a transmembrane domain and the outer secreted layer, which is continuously digested and washed out. This bilayer mucus structure is defined well in the stomach and the colon, whereas in the small intestine, the mucus is discontinuous, reflecting distinct physiological functions [4]. The lifetime of the secreted layer is short, often measured in minutes to hours, depending on the anatomical site [5,6].

**Figure 1:**
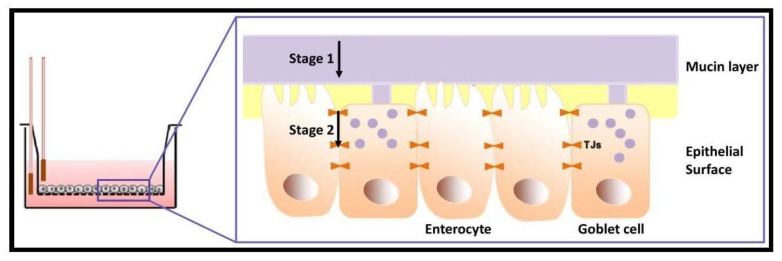
Stages of the permeability phenomenon in the in vitro model. (1) Mucin layer penetration and (2) paracellular transport across TJs. The mucin layer was only available in the co-culture system.

To exert its systemic pharmacological activity, orally administered drugs must cross the intestinal barrier formed by the intestinal mucus and the intestinal epithelium [7]. Prediction of the drug absorption plays a major role in the selection of the pharmacotherapy. Numerous in vitro, ex vivo, in vivo, and in silico models were established to evaluate the extent of drug absorption. Each method has pros and cons in terms of cost, reproducibility, and reliability [8]. In the past two decades, cell-based models of different biological barriers (e.g., intestine, skin) have been developed to better predict drug absorption and, at the same time, to reduce the use of experimental animals, which is ethically questioned [9]. In this context, the Caco-2 cell line monolayer became extremely popular as an in vitro model of the intestinal epithelium due to its ability to express most morphological and functional characteristics of absorptive small intestine cells, including tight junctions (TJs) and efflux pumps of the ATP-binding cassette superfamily [8,10], presenting good correlation for the estimation of oral drug absorption in humans [11]. However, the Caco-2 cell line also displays some shortcomings. First, the cells do not express all the relevant intestinal drug-metabolizing enzymes and they overexpress TJs. Furthermore, this cell line does not account for a mucus layer, one of the main barriers opposing drug absorption in the intestinal epithelium [7]. This motivated the development of a co-culture model composed of Caco-2 and the mucin-producing HT29-MTX cell line [12]. Since HT29-MTX cells produce mucin and do not form TJs at the same level of Caco-2 cells, the Caco-2/HT29-MTX co-culture model resembles more closely the structure and function of the small intestine barrier [13,14]. Figure 1 schematizes the two-stage absorption process in the co-culture in vitro model of the small intestine where Caco-2 cells perform as enterocytes and HT29-MTX as goblet cells. 

The use of mucoadhesive nano-drug delivery systems emerged as a very appealing approach to prolong the residence of the formulation at the delivery site (e.g., small intestine), by which the bioavailability is usually increased [15,16,17,18,19]. The prolongation of the residence time relies on the interaction of the nano-drug delivery system with the mucin layer (Figure 1). Thiolated polymers, coined as Thiomers^®^ by Bernkop-Schnürch and coworkers, were introduced as a new and promising family of mucoadhesive/mucopenetrating biomaterials for drug delivery. The key property of Thiomers^®^ is that the macromolecule bears free thiol groups that can bind cysteine domains in mucin [20]. Thiolated polymers display strong mucoadhesive/mucopenetrating properties due to the formation of inter- and intramolecular disulfide bonds with cysteine domains in mucin, leading to relatively improved stability and prolonged residence time and consequently more sustained drug release [21]. 

Amphiphilic nanocarriers (e.g., polymeric micelles) are formed by the self-assembly of amphiphilic block or graft copolymers in aqueous medium [22] and they have shown great potential for oral drug delivery [23,24,25]*.* However, they tend to disassemble upon extreme dilution. Aiming to physically stabilize amphiphilic nanocarriers by means of drug-compatible chemical pathways, we recently introduced a novel mucoadhesive nanogel produced by the self-assembly of amphiphilic chitosan (CS) graft copolymers synthesized by the hydrophobization of the side-chain with oligo(*N*-isopropylacrylamide) (oligo(NiPAAm)) blocks and non-covalent crosslinking with sodium tripolyphosphate (TPP) [26]. CS-*g*-oligo(NiPAAm) nanogels were engineered to preserve the intrinsic mucoadhesiveness of CS and its ability to transiently open TJs in the intestinal epithelium [27,28]. Preliminary permeability studies in vitro showed that the non-crosslinked counterparts cross the Caco-2 cell monolayer model [29]. However, oligo(NiPAAm) is thermo-responsive and thus, self-assembly is achieved only above its lower critical solution temperature (30–32 °C) [26]. 

Aiming to increase the aggregation tendency of CS-based amphiphiles and to gain insight into the pathways governing permeability in the small intestine, in this work, we synthesized for the first time CS-*g*-poly(methyl methacrylate) (CS-*g*-PMMA) nanoparticles that were thiolated by the conjugation of *N*-acetyl cysteine (NAC). PMMA is a biocompatible and Food and Drug Administration (FDA)-approved polymer widely used in different biomedical applications [30], including oral drug delivery systems [31,32,33]. After confirmation that both non-crosslinked and crosslinked nanoparticles display very good cell compatibility in Caco-2 and HT29-MTX cell lines in the 0.05–0.1% *w/v* concentration range, we compared the effect of crosslinking, nanoparticle concentration and thiolation on their in vitro permeability utilizing a Caco-2 monoculture and a Caco-2/HT29-MTX co-culture model. 

## 2. Experimental Section

### 2.1. Materials

Low molecular weight CS (degree of deacetylation of 94%; viscosity ≤ 100 mPa.s, Glentham Life Sciences, Corsham, UK), cerium (IV) ammonium nitrate (CAN, Strem Chemicals, Inc., Newburyport, MA, USA), nitric acid 70% (Bio-Lab, Jerusalem, Israel), hydroquinone (HQ, Merck, Hohenbrunn, Germany), tetramethylethylenediamine (TEMED, Alfa Aesar, Heysham, UK) and 1-ethyl-3-(3-dimethylaminopropyl)carbodiimide (EDC) hydrochloride (Glentham Life Sciences) were used as received. MMA (99% purity, Alfa Aesar) was distilled under vacuum to remove inhibitors before use. NAC (≥99%) was purchased from Sigma-Aldrich (Saint Louis, MO, USA) and was used as received. 

### 2.2. Synthetic Methods

#### 2.2.1. Synthesis of CS-*g*-PMMA Copolymer

The CS-*g*-PMMA copolymer was produced by the free radical polymerization of MMA in water. For this, CS (0.4 g) was dissolved in HNO_3_ (0.05 M in water, 100 mL) that was degassed by sonication for 30 min. Then, a TEMED solution (0.18 mL in 50 mL of degassed water) was poured into the CS solution and purged with nitrogen for 30 min at room temperature. The reaction mixture was magnetically stirred and heated to 35 °C, and 142 μL MMA dispersed in degassed water (48 mL) was added. Finally, a CAN solution (0.66 g in 2 mL of degassed water) was added to the polymerization reaction that was carried out for 3 h at 35 °C under continuous nitrogen flow and magnetic stirring. After 3 h, the polymerization was quenched by adding 0.13 g of HQ. The reaction product was then purified by dialysis against distilled water using a regenerated cellulose dialysis membrane with molecular weight cut-off (MWCO) of 12–14 kDa (Spectra/Por^®^ 4 nominal flat width of 75 mm, diameter of 48 mm and volume/length ratio of 18 mL/cm, Spectrum Laboratories, Inc., Rancho Dominguez, CA, USA) for at 48–72 h, frozen in liquid nitrogen and freeze-dried (Labconco Free Zone 4.5 plus L Benchtop Freeze Dry System, Kansas City, MO, USA). The product was stored at 4 °C until use.

#### 2.2.2. Synthesis of Thiolated CS-*g*-PMMA Copolymer

CS-*g*-PMMA (200 mg) and NAC (600 mg) were dissolved separately in 10 mL of degassed water. The carboxylic acid moieties of NAC were activated for 20 min by the addition of EDC solution (150 mg in 10 mL degassed water). Then, the pH of the three solutions was adjusted to 4–5, mixed together, and the reaction mixture was incubated for 6 h under magnetic stirring and nitrogen flow at room temperature. The thiolated CS-*g*-PMMA copolymer was purified by dialysis employing a regenerated cellulose dialysis membrane with MWCO of 3500 Da (Cellu·Sup^®^ Membrane Filtration Products, Inc., Seguin, TX, USA). The sequence of dialysis media was the following: (i) 1 mM HCl containing 2 μM ethylenediaminetetraacetic acid (EDTA, Sigma-Aldrich); (ii) 1 mM HCl containing 1% NaCl and (iii) 0.5 mM HCl. Samples were frozen in liquid nitrogen and freeze-dried. The product was stored at 4 °C until use. 

### 2.3. Characterization Methods

#### 2.3.1. Proton Nuclear Magnetic Resonance Spectroscopy

The different products were qualitatively analyzed by Proton Nuclear Magnetic Resonance Spectroscopy (^1^H-NMR, 400-MHz Bruker^®^ Avance III High Resolution spectrometer, Bruker BioSpin GmbH, Rheinstetten, Germany) in 5% *w/v* dimethyl sulfoxide-*d6* (DMSO-*d*_6_) solution (Sigma-Aldrich) at 25 °C, using the peak of DMSO at 2.50 ppm as internal standard. The amount of PMMA in the CS-*g*-PMMA copolymer was determined by the integration of characteristic signals of each component in physical mixtures of CS:MMA of different weight ratios (0.05–10) and calculating the ratio between the integration of the characteristic signals of CS and PMMA and 2.8 and 0.8–1.0 ppm, respectively (*R*^2^ = 0.979). Then, the ratio between the relevant peaks in the copolymer sample was calculated and interpolated in the calibration curve to determine the weight percentage (% *w/w*) of the hydrophobic component. For the physical mixture, pure CS and MMA were analyzed in deuterium oxide (D_2_O, Sigma-Aldrich) with the addition of 25 μL of trifluoroacetic acid (Sigma-Aldrich). 

#### 2.3.2. Fourier Transform Infrared Spectroscopy

Fourier Transform Infrared Spectroscopy (FTIR) samples were prepared in KBr (Merck Chemicals GmbH, Darmstadt, Germany) disks and pressed to transparency. FTIR spectra were recorded in an Equinox 55 spectrometer (Bruker Optics Inc., Ettlingen, Germany) from 4000 to 400 cm^−1^ (32–64 scans with a resolution of 4 cm^−1^) at room temperature.

#### 2.3.3. Time-of-Flight Secondary Ion Mass Spectrometry (ToF-SIMS)

Pristine and modified copolymers were analyzed by time-of-flight secondary ion mass spectrometry (ToF-SIMS, ToF-SIMS5, ION-TOF GmbH, Münster, Germany). The measurements were carried out under vacuum of 3 × 10^−9^ mBar. Secondary ions from the samples were generated using Bi+ beam with 15 keV and current of 1 pA. Spectra were measured in negative mode for an area of 100 μm × 100 μm.

#### 2.3.4. Determination of NAC Content

The degree of thiolation was determined spectrophotometrically using the Ellman’s colorimetric assay [34]. First, 0.5–1.5 mg of unmodified or thiolated copolymer was hydrated in 500 μL of 0.5 M phosphate buffer pH 8.0 and then 500 μL of Ellman’s reagent was added; this reagent is composed of 3 mg of 5,5-dithiobis(2-nitrobenzoic acid) (DTNB, Sigma-Aldrich) dissolved in 10 mL of 0.5 M phosphate buffer of pH 8.0. Samples were incubated for 2 h at room temperature. Thereafter, 200 μL of each sample was transferred to a 96-well plate and the absorbance was measured at 450 nm in a microplate reader (Multiskan GO Microplate Spectrophotometer, Thermo Fisher Scientific Oy, Vantaa, Finland). Pure NAC was used to build a calibration curve in the 10–1000 μM range (*R*^2^ = 0.993) and then, the conjugation extent was calculated by interpolating the absorbance of the corresponding thiolated copolymer in the curve. The unmodified copolymer was used as blank. 

#### 2.3.5. Self-Assembly

The critical micellar concentration (CMC) of the unmodified and the thiolated CS-*g*-PMMA copolymer in water was determined at 25 and 37 °C using dynamic light scattering (DLS, Zetasizer Nano-ZS, Malvern Instruments, Malvern, UK) at a scattering angle of 173°. Data was analyzed using CONTIN algorithms (Malvern Instruments). For this, a stock aqueous solution (0.1% *w/v* in water) of each copolymer was prepared by direct dissolution, diluted in the same medium (0.01–0.1% *w/v*), and incubated at 25 and 37 °C (overnight) to allow aggregation. Then, the intensity of the scattered light (DCR) expressed in kilo counts per second (kcps) was measured and plotted as a function of the copolymer concentration (% *w/v*). The CMC was established from the intersection of the two straight scattering lines before and after the micellization occurs. Each DLS measurement is the result of at least six runs and CMC values are presented as mean ± S.D. 

#### 2.3.6. Preparation of Crosslinked CS-*g*-PMMA Nanoparticles

Unmodified and thiolated CS-*g*-PMMA nanoparticles were prepared by direct dissolution of the copolymer in water (0.1% *w/v*) at room temperature and incubation (overnight) at 25 or 37 °C. For physical stabilization, nanoparticles were non-covalently crosslinked by the addition of 1% *w/v* TPP (Sigma-Aldrich) solution (10 μL of crosslinking solution per mL of 0.1% *w/v* nanoparticle dispersion).

#### 2.3.7. Size, Size Distribution, and Zeta-Potential

The size of the nanoparticles (expressed as hydrodynamic diameter, *D*_h_) and their size distribution (polydispersity index, PDI) was measured by DLS (see above) using 0.1% *w/v* dispersions, both at 25 and 37 °C. Zeta-potential (Z-potential) measurements required the use of laser Doppler micro-electrophoresis in the Zetasizer Nano-ZS. Each value obtained is expressed as mean ± S.D. of at least three independent samples, while each DLS or Z-potential measurement is an average of at least seven runs.

#### 2.3.8. Cell Compatibility of the Copolymers In Vitro

The compatibility of unmodified and thiolated CS-*g*-PMMA nanoparticles before and after non-covalent crosslinking with TPP was assessed in Caco-2 and HT29-MTX cell line monocultures and their co-culture in a Caco-2:HT29-MTX cell ratio of 9:1.

##### Caco-2 Cell Line

Caco-2 cell line (ATCC^®^ HTB-37TM, American Type Culture Collection, Manassas, VA, USA) was cultured in Dulbecco’s Modified Eagle’s Medium (DMEM, Life Technologies Corp., Carlsbad, CA, USA) supplemented with l-glutamine, 10% heat-inactivated fetal bovine serum (FBS, Sigma-Aldrich), and a penicillin/streptomycin antibiotic mixture (5 mL of a commercial mixture of 100 U per mL penicillin + 100 μg per mL streptomycin per 500 mL medium, Sigma-Aldrich) and incubated at 37 °C in humidified 5% CO_2_ atmosphere and split every 4–5 days. Cells were harvested with trypsin-EDTA (0.25% *w/v*, Sigma-Aldrich), and the number of live cells was counted by the trypan blue (0.4%, Alfa Aesar) exclusion assay. To assess the compatibility of the nanoparticles, cells were grown in 96-well plates (7.5 × 10^3^ cells/well, 96 h) and maintained as described above. Then, the culture medium was replaced by fresh medium (180 μL), and the sample (20 μL, 0.5% or 1% *w/v* in PBS of pH 7.4) was added to result in final nanoparticle concentrations of 0.05% and 0.1% *w/v*, respectively. After 4 and 24 h, the medium was removed, and new medium (100 μL) and sterile 3-(4,5-dimethylthiazol-2-yl)-2,5-diphenyltetrazolium bromide solution (MTT, 25 μL, 5 mg/mL, Sigma-Aldrich) was added. Samples were incubated for 3 h at 37 °C under 5% CO_2_ atmosphere, the supernatant was removed, the formazan crystals were dissolved with DMSO (100 μL), and the absorbance measured at 530 nm with reference at 670 nm (Multiskan GO Microplate Spectrophotometer). The percentage of live cells was calculated with respect to a control of cells incubated only with culture medium that was considered 100% viability. A similar assay was conducted with crosslinked unmodified and thiolated CS-*g*-PMMA nanoparticles. For this, 0.5% and 1% *w/v* copolymer solutions in PBS (pH 7.4) were incubated overnight at 37 °C and diluted 10 times with culture medium. Then, the required amount of 1% *w/v* TPP solution for crosslinking was added (see above). Finally, the culture medium was replaced by 200 μL of 0.05% and 0.1% *w/v* nanoparticle samples and cell incubated for 4 and 24 h. Results are expressed as mean ± S.D.

##### HT29-MTX Cell Line

The cell compatibility of unmodified and thiolated CS-*g*-PMMA nanoparticles before and after TPP crosslinking was evaluated in the HT29-MTX cell line (kindly donated by Prof. Bruno Sarmento from the Instituto de Engenharia Biomédica, Porto, Portugal). Cells were cultured in 96-well plates (3.5 × 10^3^ cells/well) and allowed to attach for 96 h. The MTT assay was performed as described above for Caco-2 cells.

##### Cell Compatibility in a Co-Culture of Caco-2 and HT29-MTX Cell Lines

The cell compatibility of unmodified and thiolated CS-*g*-PMMA nanoparticles before and after crosslinking was also estimated in a co-culture of Caco-2 and HT29-MTX cells. For this, cells were cultured in 96-well plates (7.5 × 10^3^ cells/well) in a Caco-2:HT29-MTX cell number ratio of 9:1 and allowed to attach for 96 h. The MTT assay was performed as described for Caco-2 cells (see above).

#### 2.3.9. Mucin Staining

For mucin staining, HT29-MTX cells in monoculture and co-culture were handled in the same manner as for the cell compatibility assays, until the stage of MTT addition when the culture medium was removed, and cells were rinsed once with PBS (pH = 7.4). Afterwards, cells were fixed with 4% paraformaldehyde (PFA, Sigma-Aldrich) in PBS for 30 min (37 °C, 5% CO_2_), PFA was removed and cells were rinsed with PBS (pH = 7.4). Cells were stained with Alcian Blue (200 μL, 1% *w/v* in 3% *v/v* acetic acid, adjusted to pH 2.5, Fluka, Deisenhofen, Germany) for 20 min at RT. Finally, the Alcian Blue solution was removed, and the cells rinsed twice with PBS (pH = 7.4) and visualized under the optical microscope (Eclipse TS100 inverted fluorescent microscope, Nikon, Tokyo, Japan).

#### 2.3.10. Permeability Studies

The apparent permeability of unmodified and thiolated CS-*g*-PMMA nanoparticles before and after TPP-crosslinking was evaluated in Caco-2 cell monolayers and Caco-2/HT29-MTX co-culture systems. For this, unmodified CS-*g*-PMMA copolymer was initially labeled (red fluorescence) with rhodamine B isothiocyanate (RITC, Sigma-Aldrich). Briefly, CS-*g*-PMMA (100 mg) was dissolved in acidic distilled water (10 mL, pH of the water was adjusted to 5.5 using acetic acid) under magnetic stirring. After complete dissolution, 10 mL of methanol was added. Then, RITC was dissolved in methanol (2.0 mg/mL, 3.5 mL), added to the copolymer solution and the mixture magnetically stirred for 3 h protected from light, at room temperature. Finally, the product was dialyzed (48 h) using regenerated cellulose dialysis membranes (MWCO of 3500 Da) to remove unconjugated RITC, frozen in liquid nitrogen and freeze-dried (see above).

Transport experiments were performed 10–25 days post-seeding of Caco-2 cells monoculture or Caco-2:HT29-MTX co-culture (9:1 cell number ratio) in cell culture inserts (ThinCert™, culture surface of 113.1 mm^2^, 3.0 μm pore size, Greiner Bio-One GmbH, Frickenhausen, Germany). Cells were maintained in 12-well plates (15.85 mm diameter, 16.25 mm height, Greiner CELLSTAR, Monroe, NC, USA) with 0.5 and 1.5 mL of DMEM medium (see above) in the apical and basolateral compartment, respectively. The total amount of cells was always 3 × 10^5^ cells per well. The culture medium was replaced every 2–3 days and the integrity of the cell monolayer was characterized by transepithelial electrical resistance (TEER) measurements performed with an epithelial volt-ohm-meter (“EVOM2”, WPI, Sarasota, FL, USA). For these experiments, only inserts where the resistance was >200 Ω·cm^−2^ were used.

Samples for the transport experiment were prepared as follows: RITC-labeled CS-*g*-PMMA was dissolved in acidic distilled water (see above) in concentration of 0.1% *w/v*. In addition, unmodified or thiolated CS-*g*-PMMA was dissolved separately in acidic distilled water in concentration of 0.9% *w/v*. To reach a final copolymer concentration of 0.1% *w/v* (and a weight ratio of unlabeled:labeled copolymer of 9:1), 0.5 mL of unlabeled and labeled CS-*g*-PMMA solution were mixed and the final volume was adjusted to 5 mL using Hank’s Balanced Salt Solution (HBSS, Sigma-Aldrich) buffered to pH 7.2 with 25 mM 4-(2-hydroxyethyl)-1-piperazineethanesulfonic acid (HEPES, Sigma-Aldrich) that was used as transport medium. Then, samples were incubated at 37 °C for at least 6 h to allow the formation of the nanoparticles. For crosslinked nanoparticles, 1% *w/v* TPP (dissolved in HBSS) was added (see above) at least 6 h after sample preparation, and then incubated at 37 °C overnight. Samples were diluted to the relevant concentration before the experiment.

At the beginning of the experiment, the medium in the apical and basolateral was replaced with transport medium (HBSS) and incubated for 15 min at 37 °C in a humidified 5% CO_2_ atmosphere. Then, transport medium in the donor (apical) compartment was replaced by the corresponding sample (0.4 mL) and in the acceptor compartment (basolateral) by fresh transport medium (1.2 mL). After 5, 10, 15, 30, 45, 60, 90, 120, 180, and 240 min, 600 μL was extracted from the basolateral compartment for quantification of the transported copolymers by fluorescence spectrophotometry (Fluoroskan Ascent Plate Reader, Thermo Fisher Scientific Oy) using black 96-well flat bottom plates (Greiner Bio-One, Kremsmünster, Austria) at wavelengths of 485 nm for excitation and 635 nm for emission. The red fluorescence in the acceptor chamber due to the permeability of RITC-labeled nanoparticles was measured and interpolated in a calibration curve built with different nanoparticle concentrations (in the 0.0001–0.1% *w/v* range) containing always a 9:1 weight ratio of unlabeled (unmodified or thiolated):labeled copolymer (*R*^2^ = 0.999). At the end of the experiment (240 min), 50 μL was also removed from the apical side of each sample in order to calculate the mass balance. The *P*_app_ was calculated according to Equation (1)

(1) $$P_{app}=\frac{dc}{dt}\cdot \frac{1}{A\cdot C_{0}}\;\left[cm\cdot s^{-1}\right]$$

where  dc/dt is the transport rate (μg/s) across the monolayer, $C_{0}$ is the initial concentration in the donor compartment ($\mu g/{\mathrm{cm}}^{3}$), and A. is the surface area of the membrane (${\mathrm{cm}}^{2}$).

#### 2.3.11. Statistical Analysis

Statistical analysis of permeability experiments was performed by *t*-test on raw data (Excel, Microsoft Office 2013, Microsoft Corporation, Redmond, WA, USA). First, the analysis was performed for *P*_app_ obtained from crosslinked systems. *p*-values were calculated between different concentration (0.05% and 0.01% *w/v*) at the same monolayer and between the same concentration in different monolayer (Caco-2 and co-culture). In addition, a further analysis was performed, and *p*-value was calculated between non-crosslinked and crosslinked systems (0.05% *w/v*).

## 3. Results and Discussion

### 3.1. Synthesis of CS-g-PMMA Copolymer

**Figure 2:**
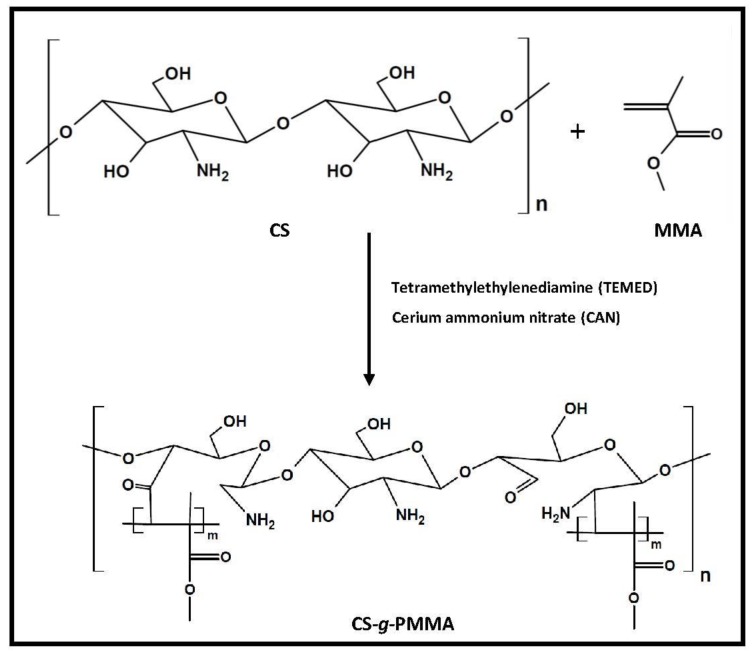
Synthetic pathway of CS-*g*-PMMA by free radical polymerization of MMA in the presence of CAN.

The synthesis of the CS-*g*-PMMA copolymer was carried out by the thermal free radical polymerization of MMA in nitric acid using CAN as initiator [26,35,36]. Both reactive –OH and –NH_2_ groups may form a complex with Ce(IV) cation, which will dissociate and create free radical sites on the CS backbone for future attack of the double bond of MMA and polymerization to render the amphiphilic derivative (Figure 2). 

**Figure 3:**
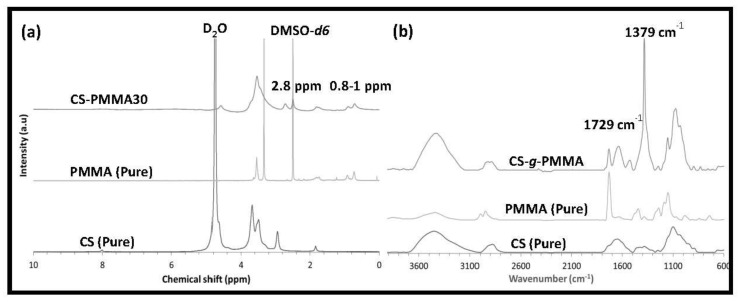
Chemical characterization of the CS-*g*-PMMA copolymer. (**a**) ^1^H-NMR spectra of pure CS, pristine PMMA ,and CS-*g*-PMMA copolymer and (**b**) FTIR spectra of pure CS, pristine PMMA and CS-*g*-PMMA copolymer.

^1^H-NMR analysis of pure CS showed a characteristic pattern with signals at 2.8 ppm (HC–NH_2_) and 3.0–4.0 ppm (methylene on the backbone), while pure PMMA presented peaks at 1.5–2.0 ppm (C–CH_2_–), 3.7 ppm (COO–CH_3_) and 0.8–1.0 ppm (C–CH_3_) (Figure 3a). 

As expected, CS-*g*-PMMA showed a combination of both spectra. It is important to mention that PMMA and the different copolymers were dissolved in DMSO-*d_6_*, while pure CS was dissolved in D_2_O with a small volume of trifluoroacetic acid because it is insoluble in DMSO. ^1^H-NMR was also used to determine that the weight percentage of PMMA (% *w/w*) in the copolymer was 30% *w/w* (see Section 2.3.1). 

The graft copolymer was also characterized by FTIR, and the spectrum was compared to those of pure CS and PMMA (Figure 3b). Pure CS displayed typical absorption bands of O–H and N–H stretching at 3456 cm^−1^ and C–H stretching, N–H bending, and C–O–C stretching in the glycosidic bonds at 2919, 1591 and 1157 cm^−1^, respectively. A band at 1641 cm^−1^ of *N*-acetyl moiety confirmed that the CS was partly deacetylated. The graft copolymer exhibited a strong characteristic band of PMMA at 1729 cm^−1^ (carboxyl group) and several bands of CS as detailed before, including a strong characteristic CS band at 1379 cm^−1^. ^1^H-NMR and FTIR results confirmed that the successful synthesis of the CS-*g*-PMMA copolymer.

### 3.2. Synthesis and Chemical Charcterization of Thiolated CS-g-PMMA Copolymer

NAC is a mucolytic drug that reduces the viscosity of mucus. This can be explained by the ability of NAC to break the disulfide bonds of mucin in the mucus layer [37]. For example, Suk et al. showed the transport of muco-inert nanoparticles in cystic fibrosis sputum pretreated with free NAC [38]. Considering that, thiolated polymers have been reported to bind cysteine domains in mucin by the formation of S–S bonds and thus to be mucoadhesive, it is unclear whether thiolation of our amphiphilic nanoparticles with NAC will confer mucoadhesive or mucopenetrating properties.

**Figure 4:**
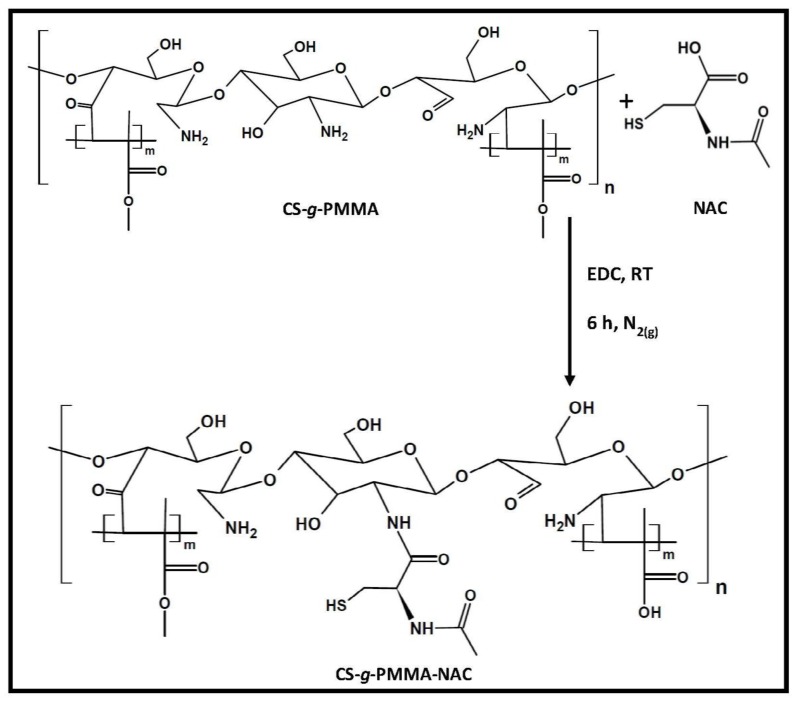
Synthetic pathway of thiolated CS-*g*-PMMA copolymer by a condensation reaction.

The thiolated CS-*g*-PMMA copolymer was synthesized by a condensation reaction between the primary amine groups of the CS backbone and the carboxylic acid group of NAC utilizing EDC as coupling agent [39] (Figure 4). 

**Figure 5:**
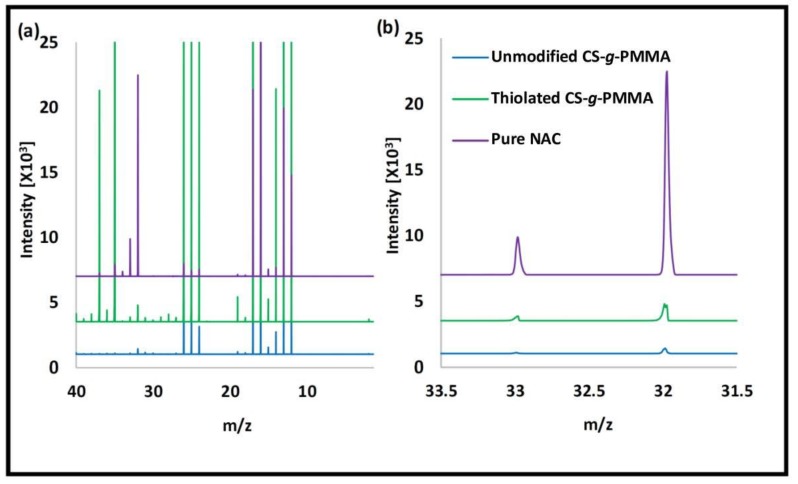
ToF-SIMS spectra of pure NAC and unmodified and thiolated CS-*g*-PMMA. (**a**) Full spectra and (**b**) Magnification of the spectra between 31.5 and 33.5 *m/z*.

Pure NAC and the unmodified and thiolated CS-*g*-PMMA copolymers were analyzed by ToF-SIMS to determine the qualitative presence of thiol (SH) due to NAC modification. Peaks in 32 and 33 *m/z* are characteristic of S and SH, respectively. NAC showed very strong peaks of S and SH (Figure 5). Unmodified CS-*g*-PMMA does not contain S in its structure. However, small S and SH contents were observed due to environmental contamination [40]. When thiolated CS-*g*-PMMA was analyzed, stronger S and SH peaks indicated the successful conjugation of NAC. To confirm that these signals in the copolymer stemmed from NAC conjugation and not from air pollution, we calculated the area ratio of these two peaks (S/SH) in the three spectra (Table 1). The area ratio of pure NAC and thiolated CS-*g*-PMMA was very similar (3.5 and 3.9, respectively) and substantially lower than that of unmodified CS-*g*-PMMA (S/SH ratio of 10.8), indicating that the presence of S and SH in thiolated CS-*g*-PMMA stemmed from the successful conjugation of NAC, as opposed to the unmodified one. 

Since ToF-SIMs was not quantitative, the concentration of thiol residues in thiolated CS-*g*-PMMA was determined by the Ellman’s colorimetric assay. According to this assay, the measured amount of thiol was 212 ± 41 μmol/g polymer (3 ± 0.7 % *w/w*). It is important to stress that SH is very sensitive to oxidation with atmospheric oxygen and formation of S–S. To reduce the oxidation extent, the reaction and the dialysis were conducted under low pH conditions [41].

### 3.3. Self-Assembly

The characterization of the self-assembly of unmodified and thiolated CS-*g*-PMMA was a fundamental aspect of the work because we envision the use of the amphiphilic nanoparticles as hydrophobic drug nanocarriers [26]. The CMC of unmodified and thiolated copolymers was measured in water at 25 and 37 °C. The former temperature is relevant for drug encapsulation under room temperature conditions, while the latter for the behavior of the nanoparticles in the biological milieu (e.g., permeability across the intestinal epithelium). The mechanism behind the self-aggregation of most amphiphilic copolymers is entropy-driven and mainly related to the release of water hydration molecules from the hydrophobic block, in this case PMMA [42]. The CMC of unmodified and thiolated CS-*g*-PMMA at 25 °C was 0.05% *w/v* (Table 2). At 37 °C, the NAC-modified copolymer showed a slight decrease of the CMC to 0.04% *w/v*.

Using 0.1% *w/v* suspensions, the size, size distribution, and Z-potential of the unmodified and thiolated CS-*g*-PMMA nanoparticles were characterized by DLS, before and after non-covalent crosslinking with 1% *w/v* TPP. Generally, the size range for all the nanoparticles was 100–330 nm (Table 3). This size range would fit the mesh size of the porous mucus layer. Regardless of the temperature, crosslinking resulted in a size growth due to the formation of intra-micellar bonds [26]. It is worth stressing that unmodified CS-*g*-PMMA nanoparticles showed a monomodal size distribution before and after the crosslinking. Conversely, the thiolated derivative showed a bimodal aggregation pattern with a major size population of 155–156 nm and a minor size population of 25–28 nm. These results suggested that regardless of the similar aggregation trend (as expressed by the unchanged CMC), thiolation slightly modified the aggregation pattern. Upon crosslinking, the size of thiolated nanoparticles became monomodal. The surface-charge of the nanoparticles was estimated by means of Z-potential. As expected, all the nanoparticles showed a positively-charged surface consistent with the presence of protonated free amine groups of CS and the polycationic nature of the hydrophilic domain (Table 3). Thiolation and crosslinking did not affect this property in a very substantial manner. This is a relevant feature towards the study of cell compatibility and permeability using in vitro cell models because on one hand, positively-charged moieties are cytototxic and, on the other, they are involved in the opening of TJs. 

### 3.4. Cell Compatibility of the Copolymers In Vitro

**Figure 6:**
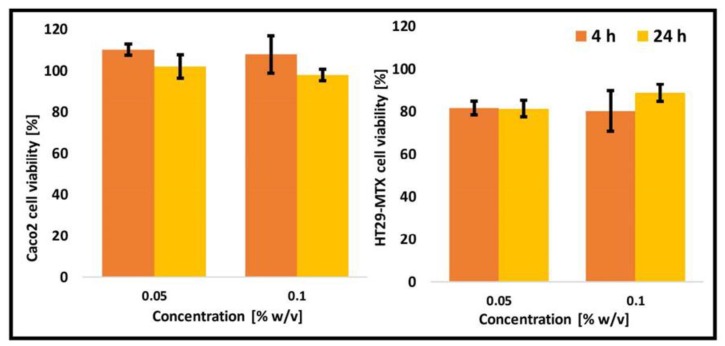
Cell viability of Caco-2 and HT29-MTX cells upon exposure to 0.05% and 0.1% *w/v* non-crosslinked unmodified CS-*g*-PMMA nanoparticles, as estimated by MTT.

In vitro cell compatibility is required to optimize the conditions for the permeability studies where the nanoparticle concentration has to ensure high compatibility. Otherwise, the permeability of the nanoparticles could stem from the generation of empty spaces in the cell monolayer and their direct interaction and crossing of the semipermeable membrane. In this context, the cell compatibility of unmodified and thiolated CS-*g*-PMMA copolymers using two cell types separately, Caco-2 and HT29-MTX, and their co-culture in a Caco-2:HT29-MTX cell number ratio of 9:1 was characterized. Initially, cells (Caco-2 and HT29-MTX) were exposed to two concentrations of unmodified CS-*g*-PMMA (0.05% and 0.1% *w/v*) and the number of cells after 4 and 24 h quantified by the MTT assay. Untreated cells were considered 100% viability and used as control. It is important to stress that permeability studies are conducted over 4 h. Both cell monolayers showed very high viability (>80%) (Figure 6), which is consistent with very good cell compatibility in vitro [43].

**Figure 7:**
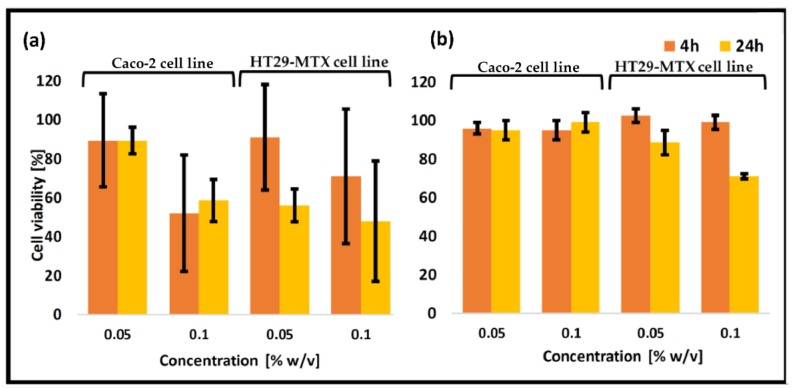
Cell viability of Caco-2 and HT29-MTX cells upon exposure to different concentrations of (**a**) non-crosslinked thiolated CS-*g*-PMMA nanoparticles and (**b**) crosslinked thiolated CS-*g*-PMMA nanoparticles, as estimated by MTT*.*

Next, Caco-2 and HT29-MTX cells were exposed to non-crosslinked thiolated CS-*g*-PMMA (0.05% and 0.1% *w/v*) and the number of live cells quantified. After 4 h, a very slight viability loss was observed for 0.05% *w/v* samples (Figure 7a). A higher concentration (0.1% *w/v*) led to a higher viability loss. Results were very similar for both cell lines in monoculture. As expected, after 24 h, a further decrease in the cell viability was observed. The increase of the cell viability loss for a more concentrated sample most probably stemmed from the cytotoxic effect of positively-charged surface of amine groups of CS [44]. Protonated amine groups can bind to the negatively charged cell membrane in a non-specific manner and cause cell toxicity both in vitro and in vivo [45,46]. For non-crosslinked thiolated nanoparticles, the positive charge was higher (Table 3), resulting in a greater viability loss (compared to unmodified nanoparticles) [47].

The cell compatibility was also measured for non-covalently crosslinked thiolated CS-*g*-PMMA nanoparticles. Remarkably, crosslinking had a very beneficial effect on cell compatibility, leading to a remarkable increase of the number of viable cells, regardless of the concentration and the exposure time (Figure 7b). For instance, after 4 h, all the nanoparticles showed viability >90%, and the values remained always >70% which is acceptable according to the ISO 10993-5 (Tests for in vitro cytotoxicity) [43]. The explanation for this improvement in cell compatibility is the partial neutralization of cytotoxic amine group after the crosslinking with TPP, as previously shown by Menaker Raskin et al. [26]. 

**Figure 8:**
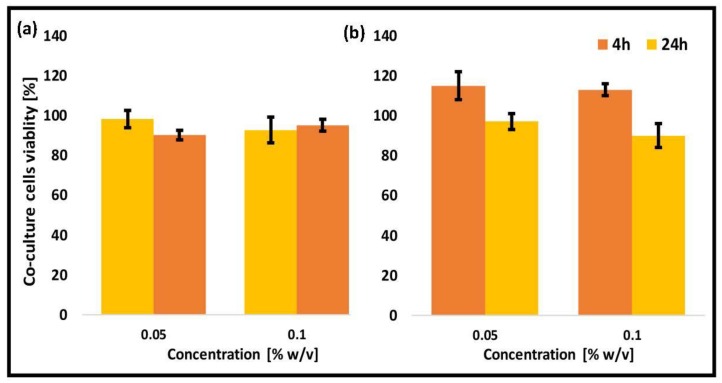
Cell viability of a Caco-2:HT29-MTX (9:1) co-culture model upon exposure to 0.05% and 0.1% *w/v* (**a**) non-crosslinked unmodified CS-*g*-PMMA nanoparticles and (**b**) crosslinked thiolated CS-g-PMMA nanoparticles, as estimated by MTT.

Caco-2 cell monoculture was extensively used to mimic the human intestinal barrier. However, it exhibits a number of limitations such as lack of mucin layer and overexpression of TJs. In this work, we used an in vitro model of intestinal barrier based on the co-culture of Caco-2:HT29-MTX cell lines in a 9:1 cell number ratio. As expected, the viability of unmodified and thiolated CS-*g*-PMMA nanoparticles was similar to the one obtained in both monocultures (Figure 8). 

Based on cell compatibility results, we decided to primarily conduct permeability studies employing nanoparticle concentrations of up to 0.05% *w/v*. At the same time, the CMC of the copolymers had to be considered, especially for non-crosslinked counterparts, as they could undergo disassembly. 

### 3.5. Mucin Staining

**Figure 9:**
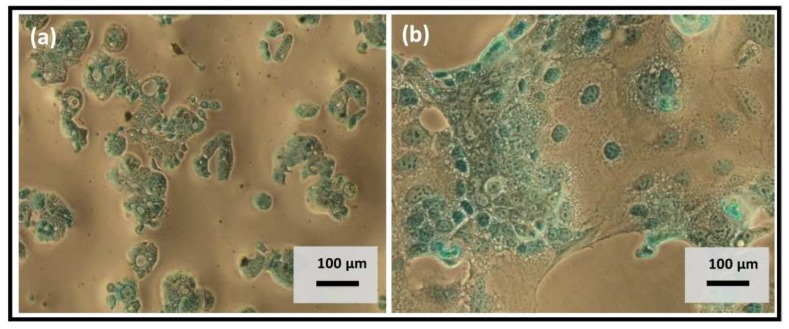
Mucin staining with Alcian Blue in (**a**) HT29-MTX monoculture and (**b**) Caco-2:HT29-MTX (9:1) co-culture.

One of the most significant advantages of using co-culture of Caco-2 and HT29-MTX as a model of the intestinal barrier is the presence of the mucin layer that allows a more reliable evaluation of the permeability features of the human intestinal barrier. To qualitatively determine that the HT29-MTX cell line produced mucin in both monoculture and co-culture with Caco-2, the Alcian Blue staining method was used. This dye is widely used to visualize mucopolysaccharides in general and acidic mucins in particular [48]. In both cases, HT29-MTX cell line showed production of acidic mucin (Figure 9).

### 3.6. Permeability Studies

The permeability of unmodified and thiolated CS-*g*-PMMA nanoparticles before and after crosslinking was evaluated in two in vitro models of the intestinal barrier: a monolayer of Caco-2 cells and a co-culture monolayer of Caco-2 and HT29-MTX cells. A comparison of the results between both models allowed us to elucidate the influence of mucin on the in vitro permeability of the nanoparticles. In addition, aiming to study the effect of nanoparticle concentration on the permeability in vitro, we conducted the same assay using crosslinked nanoparticles in two concentrations, namely 0.05% and 0.01% *w/v*. It is worth stressing that usually, this parameter is neglected in the scientific literature [49,50]. The use of non-crosslinked 0.01% *w/v* systems was precluded because this concentration is below the CMC of the copolymer (0.04–0.05% *w/v*) and thus, the nanoparticles disassemble. In the case of crosslinked samples, nanoparticles were crosslinked at a higher concentration (0.1% *w/v*) and only then diluted to the final 0.01% *w/v* concentration. 

To understand the influence of the mucin produced by HT29-MTX cells on the permeability of the nanoparticles, we compared the permeability of each one of the nanoparticles in both models. When more concentrated dispersions (0.05% *w/v*) were used, *P*_app_ values did not change (Table 4), regardless of the nanoparticle and the model (Caco-2 monoculture or Caco-2/HT29-MTX co-culture), suggesting the saturation of transport mechanisms. When a more diluted 0.01% *w/v* dispersion was used, we observed a decrease in the *P*_app_ of thiolated CS*-g-*PMMA-NAC from approximately 3.5 × 10^−6^ cm/s in Caco-2 monolayer to approximately 2.1 × 10^−6^ cm/s in a co-culture system. This change was statistically significant (*p* < 0.05) and likely stemmed from the covalent binding of the nanoparticles to the cysteine domains in mucin. This hypothesis is supported by the fact that these nanoparticles showed almost identical permeability to the unmodified counterpart when they were assessed in the Caco-2 cell monoculture model that did not present mucin.

*P*_app_ differences between the thiolated and unmodified 0.01% *w/v* nanoparticles in Caco-2 cell monolayers were not statistically significant (*p* < 0.05). In contrast, 0.01% *w/v* crosslinked unmodified nanoparticles showed an increase of the *P*_app_ in the co-culture system with respect to the Caco-2 monoculture, though differences were not statistically significant. On one hand, these results were unexpected because mucin was anticipated to hinder the permeability of nanoparticles. On the other, the interaction of CS domains in the nanoparticle with mucin is of electrostatic nature and weaker than that of NAC, that is covalent. Thus, it is likely that this interaction favored the entrapment of the nanoparticles in the mucin-containing monolayer, increasing their effective concentration on the epithelial surface and their availability to cross the monolayer with respect to a mucin-free cell monolayer. Remarkably, our findings indicated that CS-based nanoparticles could permeate a mucin-containing cell monolayer in vitro and highlight their potential for transmucosal oral drug delivery. 

NAC is a mucolytic agent that is capable of breaking the disulfide bond of the mucin glycoproteins, creating a local cleavage in the mucus layer that allows enhanced permeability. However, at the same time, NAC could form covalent binds with S–S groups in mucus [51]. In this work, we demonstrated that conjugated NAC increased the covalent interaction of the nanoparticles with mucin, at least in the investigated concentration, resulting in a decrease of the permeability in vitro. In addition, *P*_app_ values were in good agreement with previous studies conducted with non-crosslinked protoporphyrin-modified CS-*g*-poly(NiPAAm) nanoparticles [29] and were consistent with moderate and low permeability for 0.01% and 0.05% *w/v* systems, respectively [52]. Again, our results stress the relevance of the nanoparticle concentration during the permeability assay. Similar results were recently reported by Liu et al. utilizing lipid nanocarriers surface-modified with CS-NAC [53]. At the same time, it is noteworthy that based on our results, the effect of higher NAC modification extent on permeability could not be anticipated and the presence of paradoxical effects such as increased mucopenetration due to disruption of S–S domains in mucin at lower or higher NAC contents could not be ruled out. 

Next, we assessed the effect of nanoparticle concentration on the *P*_app_. Regardless of the model (monoculture or co-culture), the higher the nanoparticle concentration, the lower the *P*_app_ for all the nanoparticles (Table 4). This could be explained, as mention above, by the saturation of the transport mechanisms in the monolayer surface and the decrease of the *P*_app_. 

Non-covalent crosslinking of these novel self-assembly nanoparticles ensures physical stability under extreme dilution, improves their cell compatibility and will eventually enable a more controlled release of the encapsulated cargo [26]. In this work, the permeability of these non-covalently crosslinked nanoparticles was characterized for the first time. Since the CMC of these copolymers is approximately 0.05% *w/v*, to assess the permeability of non-crosslinked nanoparticles we only used 0.05% *w/v* systems; non-crosslinked 0.01% *w/v* nanoparticles would undergo disassembly. For Caco-2 monolayer, non-crosslinked unmodified and thiolated CS-*g*-PMMA nanoparticles showed significantly higher *P*_app_ values than the crosslinked counterparts (Table 4). This was observed for all the nanoparticles. The difference between the Caco-2 and co-culture monolayers could be explained by further analyzing the configuration of the permeability phenomenon, which can be divided into two main stages. The first stage is the permeability of the nanoparticle across the mucin layer, while the second is the crossing of the epithelial barrier by paracellular (by opening of the TJs between the cells), transcellular (by active endocytosis in the apical side and exocytosis in the basolateral one) or a combination of both (Figure 1). In previous studies, we demonstrated that Caco-2 cells do not internalize these CS-based amphiphilic nanoparticles [26]. In addition, we confirmed that unmodified non-crosslinked nanoparticles cross the Caco-2 cell monolayer by the transient opening of the TJs [29]. In the case of the Caco-2 cell monolayer model, the first stage is absent due to the lack of mucin. Thus, the permeability is governed by the ability of the nanoparticles to disrupt the TJs, a phenomenon that depends on the availability of free amine groups. Since the TPP-crosslinking employs free amine groups in the CS-*g*-PMMA nanoparticle, their concentration decreases and the ability of crosslinked unmodified and thiolated nanoparticles to cross the Caco-2 cell monolayer was affected, resulting in lower *P*_app_ values than the non-crosslinked counterparts (Table 4); e.g., *P*_app_ of unmodified and thiolated nanoparticles decreased from 2.087 and 2.591 × 10^−6^ cm/s before crosslinking to 1.215 and 0.921 × 10^−6^ cm/s, respectively, after it. In the co-culture model, crosslinking of unmodified nanoparticles also led to a significant (*p* < 0.05) decrease of *P*_app_ from 2.060 to 1.462 × 10^−6^ cm/s. This result indicated that for these nanoparticles, the overall permeability process is mainly governed by the opening of the TJs and that the contribution of the interaction with mucin plays a less critical role. Conversely, in the case of thiolated nanoparticles, crosslinking did not significantly alter the *P*_app_. These findings supported that thiol groups in non-crosslinked and crosslinked nanoparticles bind to mucin in a similar manner and increase the effective availability of the nanoparticles that once in the proximity of the epithelial monolayer cross it, most likely, by the paracellular route.

## 4. Conclusions

This work investigated the development of a novel type of amphiphilic nanoparticle produced by the self-assembly of a CS-*g*-PMMA copolymer before and after thiolation with NAC and its later stabilization by ionotropic crosslinking of the CS domains. The sizes of the formed pristine and thiolated nanoparticles were in the range of 100–300 nm. After crosslinking, both copolymers showed a monodisperse side distribution. Both the unmodified and thiolated copolymers displayed good cytocompatibility in Caco-2 and HT29-MTX cell lines separately and in co-culture. Crosslinking improved the cell compatibility owing to the reduction of available free primary amine groups usually associated with cell toxicity. Finally, the permeability across two in vitro models of the epithelium barrier was evaluated in Caco-2 cell line monoculture and co-culture of Caco-2:HT29-MTX cell line that produced acidic mucin. Results indicated that the nanoparticle concentration is a crucial parameter that determines the ability of the nanoparticles to penetrate the monolayer (regardless of the monolayer type). In addition, we observed that conjugated NAC in the investigated concentrations increases the interaction of the nanoparticles with mucin and decreases the permeability in vitro in the co-culture model. Moreover, crosslinking of the nanoparticles decreased the permeability due to the reduce concentration of amine groups involved in the transient opening of TJs. Overall, our results confirm the great potential of these nanoparticles for transmucosal drug delivery. Future studies will assess their performance in vivo.

## Figures and Tables

**Table 1:** Ratio the peak area of S and SH, as determined in ToF-SIMS spectra.

Sample	S/SH area ratio
NAC	3.5
Unmodified CS-*g*-PMMA	10.8
Thiolated CS-*g*-PMMA	3.9

**Table 2:** Critical micellar concentration (CMC) values of unmodified and thiolated CS-*g*-PMMA copolymers in water at 25 and 37 °C, as determined by dynamic light scattering (DLS).

Copolymer	CMC (% *w/v*)	
	25 °C	37 °C
**Unmodified CS-*g*-PMMA**	0.05	0.05
**Thiolated CS-*g*-PMMA**	0.05	0.04

**Table 3:** Size (*D*_h_), size distribution (PDI), and Z-potential of 0.1% *w/v* pristine and thiolated CS-g-PMMA nanoparticles in water at 25 and 37 °C, as measured by DLS.

Copolymer	T [°C]	Non-crosslinked nanoparticles			Crosslinked nanoparticles		
		*D*_h_ (nm) ± S.D. (Relative intensity %)	PDI	Z-Potential (mV)	*D*_h_ (nm) ± S.D. (Relative intensity %)	PDI	Z-Potential (mV)
Unmodified CS-*g*-PMMA	25	127 ± 9 (100)	0.389	+27	241 ± 11 (100)	0.192	+18
Thiolated CS-*g*-PMMA		156 ± 6 (94) 25 ± 3 (6)	0.288	+33	221 ± 11 (100)	0.237	+26
Unmodified CS-*g*-PMMA	37	184 ± 4 (100)	0.201	+25	332 ± 52 (100)	0.336	+17
Thiolated CS-*g*-PMMA		155 ± 5 (94) 28 ± 1 (6)	0.282	+37	192 ± 5 (100)	0.228	+29

**Table 4:** Apparent permeability coefficient (*P*_app_) of unmodified and thiolated nanoparticles before and after crosslinking in Caco-2 and co-culture models.

Sample	Crosslinking	Concentration (% *w/v*)	*P*_app_ ± S.D. (10^−6^ cm/s)
		**Caco-2 monolayer**	
Unmodified CS-*g*-PMMA	Yes	0.01	2.997 ± 0.455
	No	0.05	2.087 ± 0.226
	Yes	0.05	1.215 ± 0.245
Thiolated CS-*g*-PMMA	Yes	0.01	3.498 ± 0.682
	No	0.05	2.591 ± 0.160
	Yes	0.05	0.921 ± 0.399
		**Co-Culture monolayer**	
Unmodified CS-*g*-PMMA	Yes	0.01	3.660 ± 0.915
	No	0.05	2.060 ± 0.147
	Yes	0.05	1.462 ± 0.243
Thiolated CS-*g*-PMMA	Yes	0.01	2.125 ± 0.460
	No	0.05	0.713 ± 0.251
	Yes	0.05	0.912 ± 0.150
